# Tick-borne pathogens in Ixodidae ticks collected from privately-owned dogs in Italy: a country-wide molecular survey

**DOI:** 10.1186/s12917-020-2263-4

**Published:** 2020-02-07

**Authors:** Stefania Zanet, Elena Battisti, Paola Pepe, Lavinia Ciuca, Liliana Colombo, Anna Trisciuoglio, Ezio Ferroglio, Giuseppe Cringoli, Laura Rinaldi, Maria Paola Maurelli

**Affiliations:** 1 Department of Veterinary Sciences, University of Turin, largo Braccini 2, 10095 Grugliasco, TO Italy; 2 Department of Veterinary Medicine and Animal Productions, University of Naples Federico II, Via Federico Delpino, 1, 80137 Naples, Italy; 3 MSD Animal Health, Via Fratelli Cervi, 20090 Segrate, MI Italy

**Keywords:** *Anaplasma*, *Babesia*, *Borrelia*, *Ehrlichia*, Ticks, dogs

## Abstract

**Background:**

Ticks and tick-borne diseases are increasingly recognized as a cause of disease in dogs worldwide. The epidemiology of ticks and tick-transmitted protozoa and bacteria has changed due to the spread of ticks to urban and peri-urban areas and the movement of infected animals, posing new risks for animals and humans. This countrywide study reports information on distribution and prevalence of pathogens in ticks collected from privately-owned dogs in Italy.

We analyzed 2681 Ixodidae ticks, collected from 1454 pet dogs from Italy. Specific PCR protocols were used to detect *i)* Piroplasms of the genera *Babesia* and *Theileria*, *ii)* Gram-negative cocci of the family Anaplasmataceae and *iii) Borrelia burgdorferi* sensu lato. Sequencing of positive amplicons allowed for species identification.

**Results:**

*Babesia*/*Theileria* spp. DNA was detected in 435 homogeneous tick-pools (Minimum Infection Rate (MIR) = 27.6%; 95% confidence interval (CI) = 25.4–29.8%) with higher prevalence in *Ixodes ricinus and Rhipicephalus sanguneus* group. The zoonotic *B. venatorum* was the most prevalent species (MIR = 7.5%; 95% CI = 6.3–9.0%). *Anaplasma* and *Ehrlichia* species were detected in 165 tick-pools (MIR = 10.5%; 95% CI = 9.3–11.8%) and specifically, *A. phagocytophilum* was identified with MIR = 5.1% (95% CI = 4.1–6.3%). *Borrelia burgdorferi* s.l. and *B. afzelii* were detected with MIR = 0.4% (95% CI = 0.2–0.8%) and MIR = 0.3% (95% CI 0.1–0.7%) respectively.

**Conclusions:**

Zoonotic pathogens *B. venatorum* and *A. phagocytophilum* were the most frequently detected in ticks collected from privately-owned dogs which might be used as markers of pathogens presence and distribution.

## Background

Ixodid ticks (Acari: Ixodidae) are, after mosquitoes, the leading vectors of pathogens of medical and veterinary importance on a global scale [1]. They are ectoparasites of domestic and wild animals, as well as humans, and feed on vertebrate hosts to develop and reproduce. While feeding, they can transmit viruses, bacteria, protozoa and helminths that may subsequently infect the host [2]. Globally, the incidence/prevalence of tick-borne diseases is rising [3, 4], mostly due to increased interactions between pathogens, vectors and hosts. Some of the most important factors that account for the increasing incidence include urbanization and human population growth, behavioral changes such as human encroachment into natural environments, climate and habitat changes, and increased wildlife populations in urban and peri-urban areas [5, 6].

Tick-borne pathogens (TBPs) able to cause disease in humans are overwhelmingly zoonotic [7]. Domestic dogs may be infected with TBPs of sylvatic origin and are also competent reservoirs for human tick-transmitted infectious agents, such as *Ehrlichia chaffeensis*, *Ehrlichia ewingii*, and *Rickettsia conorii* [8]. Wild animals are usually considered the main reservoir hosts of TBPs like *Borrelia burgdorferi* sensu lato (s.l.), *Anaplasma phagocytophilum*, *Babesia venatorum* and *B. microti* [9–12]. Dogs provide a means by which infected ticks can be carried into domestic settings, thus enhancing the risk of human infection, and can act as “sentinels” for monitoring the risk of human disease in an endemic area [13, 14].

Several country-wide studies have been made in Europe to assess ticks and TBPs presence and distribution in companion animals [15–20]. In Italy, several efforts have been made to evaluate the prevalence of circulating tick-borne pathogens in ticks collected from dogs [21, 22], although limited to certain areas. In order to better understand the distribution of TBPs in Italy, we propose the first large-scale molecular survey on TBPs harbored in ticks collected from privately-owned dogs [23]. We selected as target TBPs protozoa of the genera *Babesia* and *Theileria*, bacteria belonging to the family Anaplasmataceae and to the *Borrelia burgdorferi* s.*L. complex*. All target TBPs were chosen for their importance in human and/or animal health.

## Results

A total of 2681 Ixodidae ticks grouped into 1578 homogeneous pools were included (Table 1). The analyzed samples originated from 1454 privately-owned dogs from 78 Italian NUTS3 provinces (hereinafter NUTS3, Nomenclature of Territorial Units for Statistics, level 3), (mean = 18.64 dogs/province, standard deviation = 24.75) and 1389 municipalities (LAU2, Local Administrative Units, level 2).

**Table 1:** Genera, species and number of ticks (plus number of homogeneous pools) per species, life stage and engorgement status included in the molecular study

Genera	Species	N. of Ticks (n. of pools)	Adults			Nymphs	Larvae
			Males	Females	Engorged females		
*Dermacentor*	*D. marginatus*	5 (2)	1 (1)	4 (1)	0	0	0
	*D. reticulatus*	7 (6)	4 (3)		3 (3)	0	0
*Haemaphysalis*	*H. punctata*	4 (3)	0	2 (2)	0	2 (1)	0
*Ixodes*	*I. canisuga*	2 (1)	0	2 (1)	0	0	0
	*I. hexagonus*	112 (96)	4 (4)	48 (41)	45 (39)	14 (11)	1 (1)
	*I. ricinus*	611 (516)	64 (34)	319 (285)	195 (172)	26 (22)	7 (3)
*Rhipicephalus*	*R. bursa*	10 (5)	3 (1)	6 (3)	0	1 (1)	0
	*R. sanguineus* group	1930 (949)	628 (236)	761 (484)	189 (122)	330 (94)	22 (13)
Total		2681 (1578)	704 (279)	1142 (817)	432 (336)	373 (129)	30 (17)

### Babesia/Theileria

DNA of protozoa belonging to the genera *Babesia* and *Theileria* was detected in 435 pools (MIR = 27.6%; 95% CI = 25.4–29.8) from 395 dogs.

**Fig. 1:**
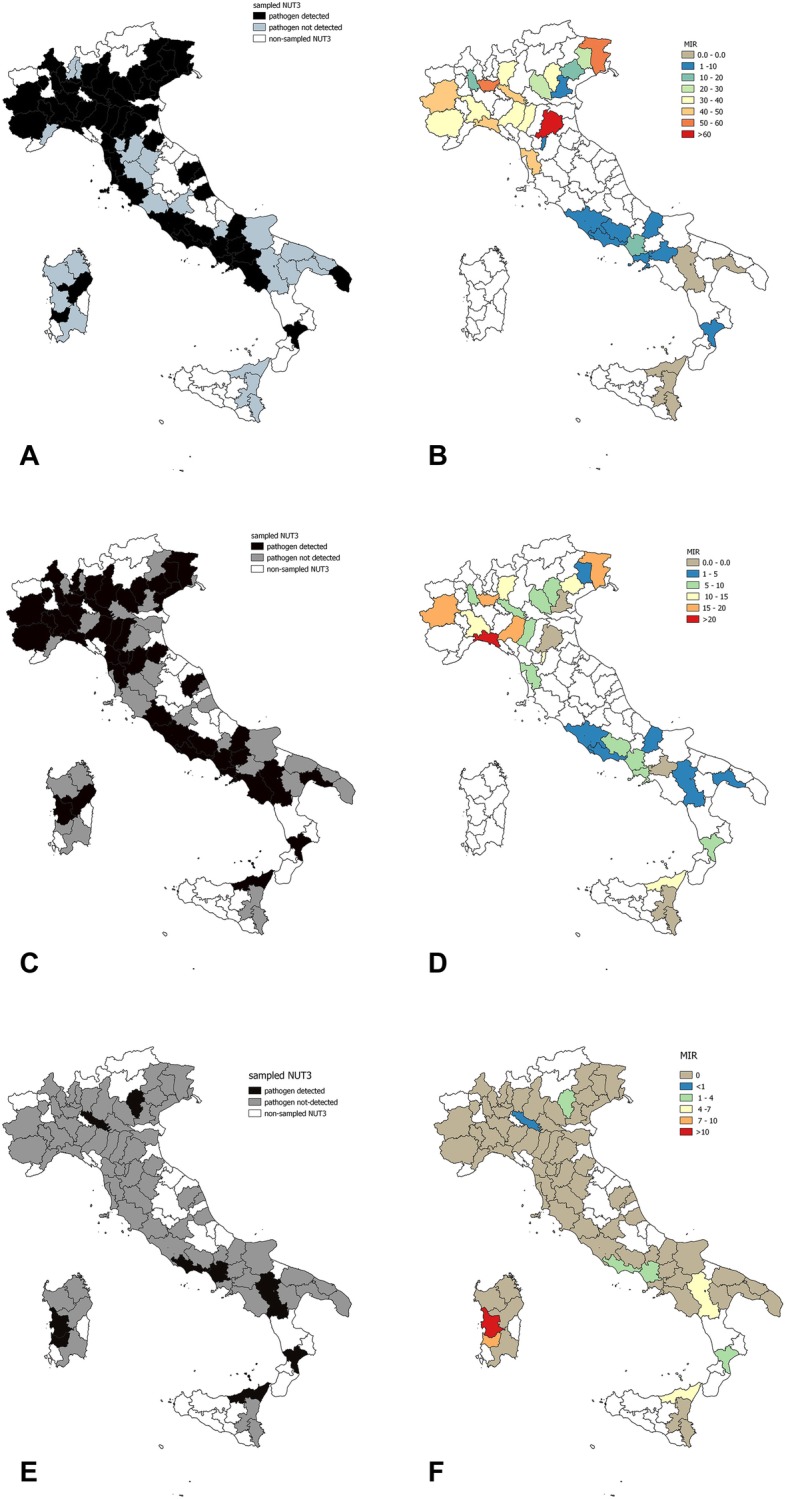
Geographical distribution, at the NUTS3 level, of ticks infected with *Babesia*/*Theileria* piroplasms (**a**) *Anaplasma*/*Ehrlichia* spp. (**c**) and *Borrelia burgdorferi* s.l. (**e**), Minimum Infection Rate (MIR%) in NUTS3 provinces where at least 20 dogs were sampled, for *Babesia*/*Theileria* (**b**), *Anaplasma*/*Ehrlichia* (**d**) and *B. burgdorferi* s.l. (**f**). Map created in QGIS 3.4.10 [24]

**Fig. 2:**
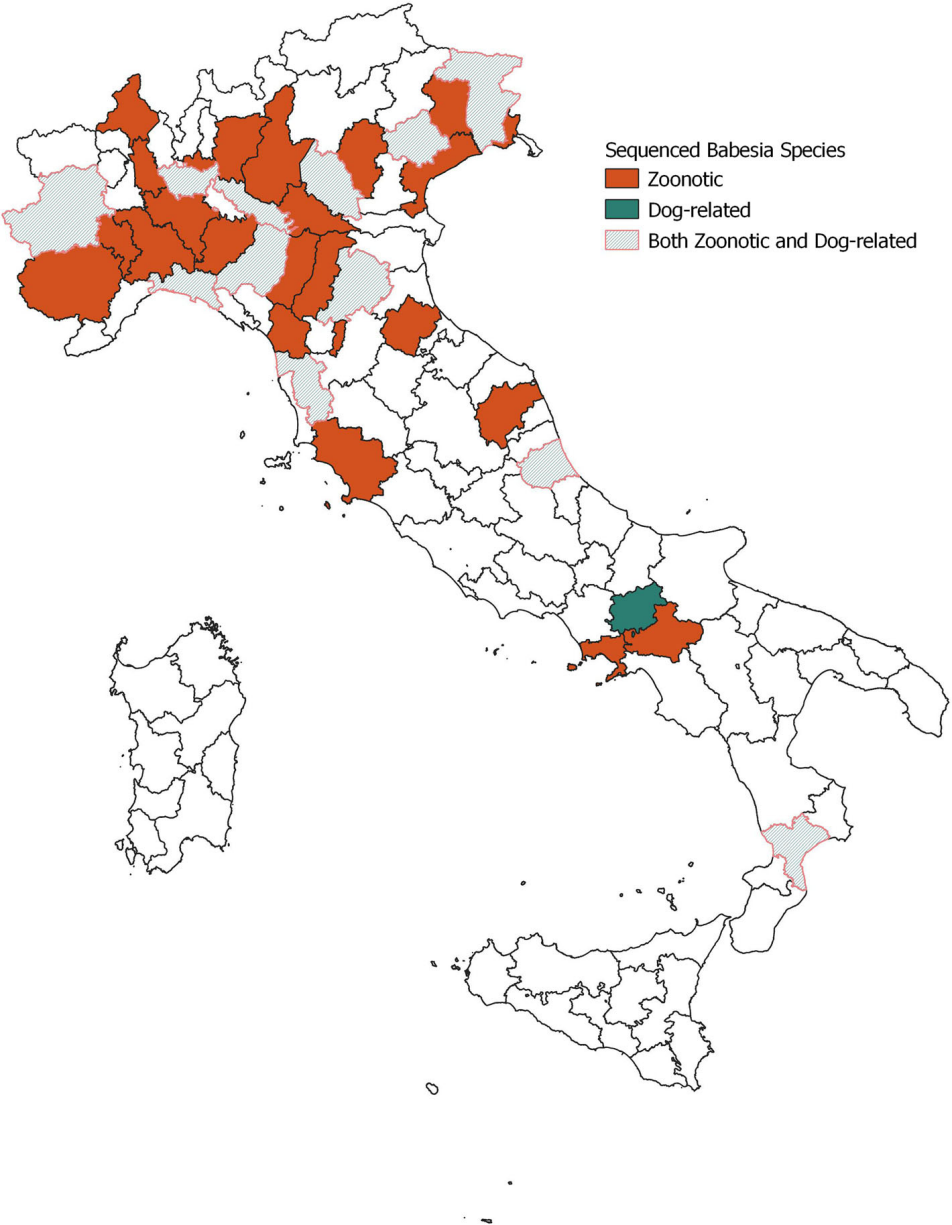
Zoonotic (*B. venatorum* and *B. microti*) and dog-related (*B. canis*, *B. vogeli* and *B. vulpes* n. sp.) *Babesia* spp. geographical distribution at NUTS3 level. Map created in QGIS 3.4.10 [24]

A significantly higher prevalence was found in *I. ricinus* (χ^2^ = 5.5, *p* = 0.02) and in ticks of the *R. sanguineus* group (χ^2^ = 4.1, *p* = 0.04) compared to other tick species as well as in adult ticks (χ^2^ = 9.99, *p* = 0.001) and engorged females (χ^2^ = 15.82, *p* = 0.000). Coinfection with Piroplasms and Anaplasmataceae was reported in 63 tick pools (*n* = 47 pools of adult *I. ricinus*, *n* = 2 pools of adult *I. hexagonous* and *n* = 11 pools of adult and n = 1 nymph pools of *R. sanguineus* group). Dogs living in urban environments were at a lower risk of carrying a *Babesia/Theileria*-infected tick (odds ratio (OR) = 0.31; 95% CI = 0.24–0.39) compared to dogs living in rural and forest habitats; housing (indoor, garden, kennel) did not influence the risk of being parasitized by an infected tick (*p* > 0.05). Breed, sex and age had not significant association with the infection status of ticks (p > 0.05). Geographical distribution at the NUTS3 level of *Babesia/Theileria-*infected ticks is reported in Fig. 1. Piroplasms were detected in 53 provinces (53/78 = 68, 95% CI = 57.0–77.2%) (Fig. 1a) with significant differences among the provinces (*p* < 0.05). Considering NUTS3 provinces where at least 20 dogs were sampled, piroplasms were detected with MIR values ranging from 0% (95% CI = 0.0–17.6%) to 61.9% (95% CI = 40.9–79.3%) (Additional file 1: Table S1, Fig. 1b). Regular antiparasitic treatment reduced the risk of being parasitized by *Babesia*/*Theileria-*positive ticks (OR = 0.24; 95% CI = 0.19–0.31). Although dogs treated with collars (OR = 6.99; 95% CI = 3.89–12.55) and spot-on products (OR = 7.75; 95% CI = 5.18–11.59) were more likely to be parasitized than those treated with oral formulations. Sequencing determined the presence of at least 9 species of the genus *Babesia* and 5 species belonging to the genus *Theileria,* as reported in Table 2. For 37 PCR-positive samples, sequencing was not possible due to low-quality DNA. The zoonotic *B. venatorum* was the most prevalent species (MIR = 7.5%; 95% CI = 6.3–9.0%), followed by unspecified *Babesia* spp. (MIR = 4.4%; 95% CI = 3.5–5.5%) and *B. capreoli* (MIR = 3.6%; 95% CI = 2.7–4.6%). Other zoonotic isolates belonged to the *B. microti* group, which were reported with MIR = 2.4% (95% CI =1.8–3.3%). For 4 tick-pools, it was possible to specifically determine the presence of *B. microti* “Munich-type” (MIR = 0.3%; 95% CI = 0.1–0.7%). Piroplasms with the domestic dog as their primary reservoir host were reported with a lower prevalence (*B. canis* MIR = 0.4, 95% CI = 0.2–0.8%; *B. vogeli* MIR = 0.6, 95% CI = 0.3–1.2%). The geographical distribution of zoonotic and dog-related piroplasms is reported in Fig. 2.

**Table 2:** Pathogen species and number of homogeneous tick pools positive for each species, Minimum Infection Rate (MIR), and MIR confidence intervals (CI) at 95% are reported below

Species	Positive Pools	*D. marginatus*	*I. canisuga*	*I. hexagonus*	*I. ricinus*	*R. sanguineus* group	*H. punctata*	Query Coverage	Max Identity	GenBank Accession Number
*B. canis*	6; (0.4%; 0.2–0.8%)					6 (0.6%; 0.3–1.4%)		100%	100%	MK571831
*B. capreoli*	56; (3.6%; 2.7–4.6%)			9 (9.4%; 5.0–16.9%)	35 (6.8%; 4.9–9.3%)	12 (1.3%; 0.7–2.2%)		100%	99–100%	KX839234
*B. capreoli/divergens*	9; (0.6%; 0.3–1.1%)				8 (1.6%; 0.8–3.0%)	1 (0.1%; 0.0–0.6%)		60–90%	87–100%	KX839234
*B. microti*	38; (2.4%; 1.8–3.3%)	1 (5%; 9.5–90.6%)		3 (3.1%; 1.1–8.8%)	12 (2.3%; 1.3–4.0%)	22 (2.3%; 1.5–3.5%)		94–100%	99–100%	MG182158 FJ608739
*B. microti*-Munich type	4; (0.3%; 0.1–0.7%)					4 (0.4%; 0.2–1.1%)		100%	100%	AB071177
*Babesia* spp.	69; (4.4%; 3.5–5.5%)		1 (100%; 20.7–100%)	3 (3.1%; 1.1–8.8%)	14 (2.7%; 1.6–4.5%)	51 (5.4%; 4.1–7.0%)		100%	100%	KJ486571/ KT182986/KY290979/ KJ486571
*B. venatorum*	119; (7.5%; 6.3–9.0%)			4 (41.7%; 1.6–10.2%)	54 (10.5%; 8.1–13.4%)	61 (6.4%; 5.0–8.2%)		100%	100%	KX857480 / MF510178
*B. vogeli*	10; (0.6%; 0.3–1.2%)					10 (1.1%; 0.6–1.9%)		100%	100%	KY290979
*B. vulpes* n. sp.	12; (0.8%; 0.4–1.3%)			3 (3.1%; 1.1–8.8%)	7 (1.4%; 0.7–2.8%)	2 (0.2%; 0.1–0.8%)		100%	98%	KT223483 / FJ608737
*T. buffeli/ sergenti/orientalis*	51; (3.2%; 2.5–4.2%)			2 (2.1%; 0.6–7.3%)	13 (2.5%; 1.5–4.3%)	36 (3.8%; 2.8–5.2%)		95–100%	98–100%	MH327771
*T. cervi*	9; (0.6%; 0.3–1.1%)				7 (1.46%; 0.7–2.8%)	2 (0.2%; 0.1–0.8%)		100%	97%	MG041373
*T. equi*	6; (0.4%; 0.2–0.8%)				4 (0.8%; 0.3–2.0%)	2 (0.2%; 0.1–0.8%)		100%	100%	KJ787768
*T. ovis*	6; (0.4%; 0.2–0.8%)				1 (0.2%; 0.0–1.1%)	5 (0.5%; 0.2–1.2%)		100%	100%	KT851432
*Theileria* spp.	3; (0.2%; 0.1–0.6%)				1 (0.2%; 0.0–1.1%)	2 (0.2%; 0.1–0.8%)		100%	97%	KF270741
*A. ovis*	3; (0.2%; 0.1–0.6%)					3 (0.3%; 0.1–0.9%)		100%	100%	MG869525
*A. phagocytophilum*	80; (5.1%; 4.1–6.3%)			4 (41.7%; 1.6–10.2%)	59 (11.4%; 9.0–14.5%)	17 (1.8%; 1.1–2.9%)		98%	100%	KY924885 / MG637125 / MH122891 / MK271308
*A. platys*	13; (0.8%; 0.5–1.4%)			1 (1.0%; 0.2–5.7%)	6 (1.2%; 0.5–2.5%)	6 (0.6%; 0.3–1.4%)		100%	100%	MH762081
*Anaplasma* spp.	36; (2.3%; 1.7–3.1%)				24 (4.7%; 3.2–6.8%)	12 (1.3%; 0.7–2.2%)		100%	100%	KY924885
*E. canis*	21; (1.3%; 0.9–2.0%)			2 (2.1%; 0.6–7.3%)	16 (3.1%; 1.9–5.0%)	2 (0.2%; 0.1–0.8%)	1 (33.3%; 6.2–79.2%)	99%	100%	KY594915
*Ehrlichia* spp.	12; (0.8%; 0.4–1.3%)			2 (2.1%; 0.6–7.3%)	8 (1.6%; 0.8–3.0%)	2 (0.2%; 0.1–0.8%)		96–98%	96–100%	MF142766 / LC120821 / AY098730
*B. afzelii*	4; (0.3%; 0.1–0.7%)			1 (1.0%; 0.2–5.7%)	3 (0.6%; 0.2–1.7%)			100%	100%	KY213885
*B. burgdorferi* s.l.	6; (0.4%; 0.2–0.8%)				1 (0.2%; 0.0–1.1%)	5 (0.5%; 0.2–1.2%)		100%	100%	KX646201

### Anaplasma/Ehrlichia

Genomic DNA of Gram-negative bacteria of the genera *Anaplasma* and *Ehrlichia* was detected in 165 tick- pools (MIR = 10.5%; 95% CI = 9.3–11.8%) from 160 dogs.

**Fig. 3:**
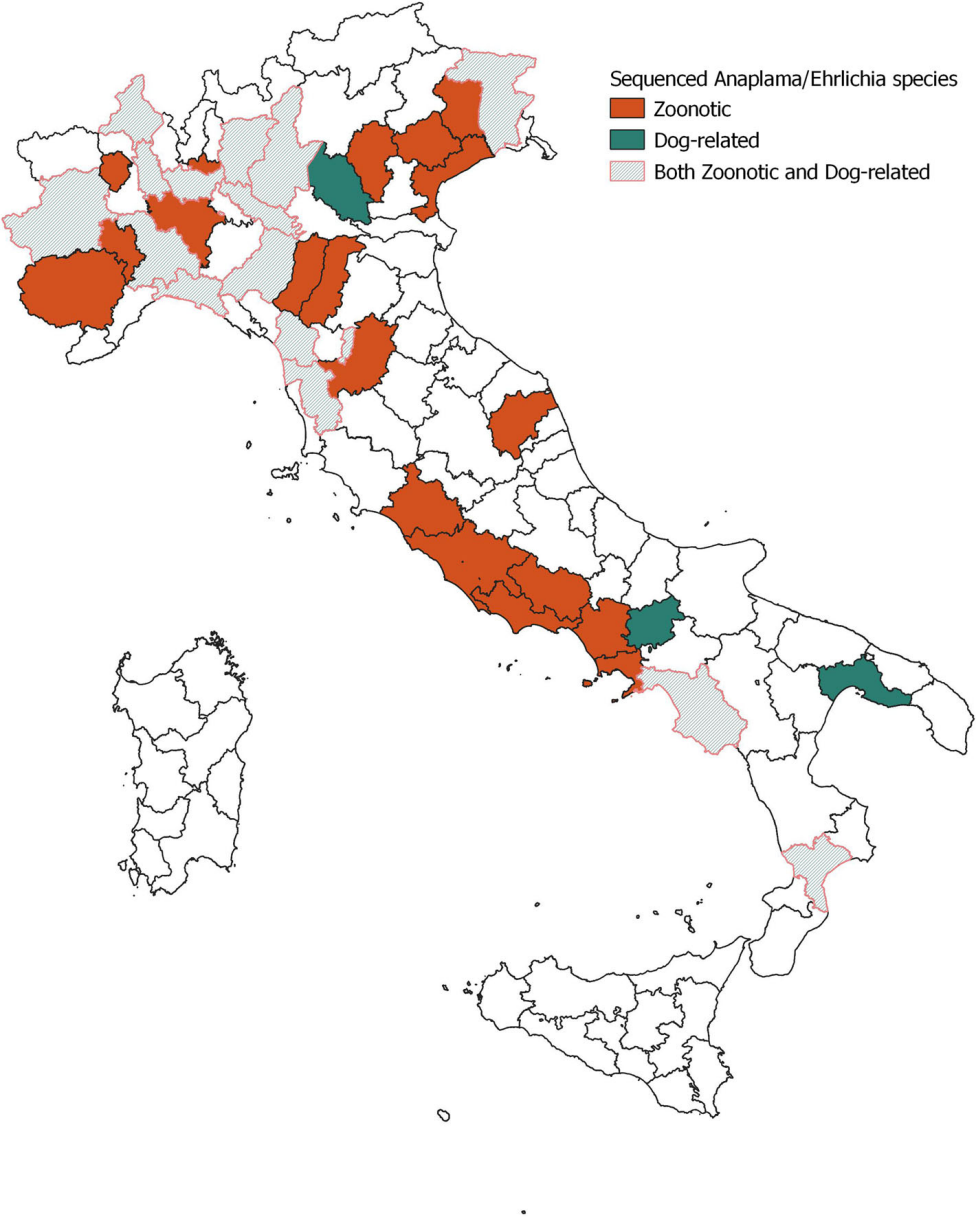
Zoonotic (*A. phagocytophilum*) and dog-related (*A. platys* and *E. canis*) *Anaplasma* and *Ehrlichia* spp. geographical distribution at NUTS3 level. Map created in QGIS 3.4.10 [24]

A higher prevalence was found in *I. ricinus* (OR = 5.33; 95% CI = 3.70–7.67), while ticks of the genus *Rhipicephalus* were significantly less infected (OR = 0.19; 95% CI = 0.13–0.27). Engorged *I. ricinus* females were more infected than other developmental stages (OR = 2.39; 95% CI = 1.48–3.53). A higher infection prevalence was found in tick-pools of dogs from forest environments compared to dogs living in only urban or rural environments (OR = 5.27; 95% CI = 3.66–7.59). Housing, breed, sex, age and the use of antiparasitic treatment had no effect on the risk of being parasitized by infected ticks (*p* > 0.05). Geographical distribution at NUTS3 level of *Anaplasma/Ehrlichia*-infected ticks is reported in Fig. 1. *Anaplasma/Ehrlichia* DNA was detected in 46 of the 78 (59%) provinces sampled (95% CI = 47.89–69.22%) (Fig. 1c) with differences between the NUTS3 provinces (*p* = 0.01). Considering NUTS3 where at least 20 dogs were sampled, *Anaplasma/Ehrlichia* DNA was detected with MIR values ranging from 0% (95% CI = 0.0–15.5%) to 22.7% (95% CI = 10.1–43.4%) (Additional file 1: Table S2, Fig. 1d). The zoonotic *A. phagocytophilum* was identified by sequencing in 80 tick-pools (MIR = 5.1, 95% CI = 4.1–6.3%) from 35 provinces, while *A. platys* and *E. canis,* which cause cyclic canine thrombocytopenia and canine monocytic ehrlichiosis, were detected in 13 (MIR = 0.8%; 95% CI = 0.5–1.4%) and 21 (MIR = 1.3%; 95% CI = 0.9–2.0%) pools respectively. *A. ovis* was detected in 3 tick-pools from Catania province (Sicily, Southern Italy) (MIR = 0.2, 95% CI = 0.1–0.6%). Uncultured *Anaplasma* spp. was amplified from 36 pools (MIR = 2.3, 95% CI = 1.7–3.1%) and uncultured *Ehrlichia* spp. from 12 pools (MIR = 0.8, 95% CI = 0.4–1.3%), including 1 isolate from northeastern Italy of *Candidatus E. walkerii* [GenBank: AY098730]*,* previously identified in *I. ricinus* ticks attached to asymptomatic human patients from the same part of Italy [25]. Table 2 reports the overall sequencing results for *Anaplasma/Ehrlichia* related to tick species. Figure 3 shows the geographical distribution of zoonotic and canine-related Anaplasmataceae (*A. platys* and *E. canis*).

### B. Burgdorferi s.l.

*B. burgdorferi* s.l. DNA was detected in 10 tick pools (MIR = 0.6, 95% CI = 0.3–1.2%) from 10 different dogs. All infected pools were comprised of adult individuals (*n* = 8 non-engorged adults and *n* = 2 engorged females). Infected pools belonged to the genus *Ixodes* (*I. ricinus n* = 4, *I. hexagonous n* = 1) and to the *R. sanguineus* group, with no statistically significant differences among genera or species due to the small number of positive samples. One fully engorged *I. ricinus* female was at the same time positive by PCR for *Anaplasma*/*Ehrlichia*. All dogs with *B. burgdorferi* s.l. positive ticks were housed indoors with access to a garden. Seven dogs regularly attended rural and forest environments, while 3 lived exclusively in an urban setting. Antiparasitic treatment was reported for 6 dogs, but active in only 2 dogs. Sequencing identified *n* = 6 *B. burgdorferi* s.l. and n = 4 *B. afzelii* (Table 2). Geographical distribution at NUTS3 level of *B. burgdorferi s.l.* is reported in Fig. 1 (cf also Additional file 1: Table S3). *B. burgdorferi s.l.* was detected in 11.5% of the sampled NUTS3 provinces (95% CI = 6.2–20.5%).

## Discussion

Ticks and tick-borne diseases have shown patterns of “general emergence” over the past few decades [26]. When pets like domestic dogs are involved, they are perceived by public opinion as a significant threat to both animal and human health [4, 7, 8]. Protozoa of the genera *Babesia*/*Theileria* were detected in 27.6% of the examined tick pools, with a higher prevalence in *I. ricinus,* which is the second most frequently reported tick affecting Italian dogs [23]. The importance of *I. ricinus* in relation to the epidemiology of *Babesia* and *Theileria* is confirmed by the large variety of species infecting this tick species. Piroplasms for which wild animals are the definitive reservoir hosts were detected with a higher prevalence in *Ixodes* species, especially the zoonotic *B. venatorum*. Given its widespread distribution, feeding habits and anthropophagic behavior, *I. ricinus* can transmit a wide variety of pathogens, linking together sylvatic, rural and peri-urban environments [27]. Notably, other zoonotic *Babesia* species, i.e. *B. microti* and *B. microti* “Munich-type”, were detected not only in I*. ricinus* but also in *R. sanguineus* group, *I. hexagonus* and *D. marginatus*. Isolates of *B. vulpes* n. sp. [28] were detected with a higher prevalence in *I. hexagonus*, but also in *I. ricinus* and *R. sanguineus* group, as previously reported [29, 30]. Clinical symptoms in dogs infected with *B. vulpes* n. sp. include pale mucous membranes, anorexia, apathy and fever with severe macrocytic/hypochromic regenerative anemia and thrombocytopenia [28, 31, 32]. Particular attention should be paid to this emergent canine pathogen, which is considered to be endemic in most European countries [33]. The lower percentage of infected tick-pools found on dogs which attend exclusively urban environments reflects the lower burden of canine piroplasms (*B. canis* and *B. vogeli*) detected only in the competent vector, *R. sanguineus* group [34]. *B. canis* was in fact detected in 0.4% of sequenced tick-pools, *B. vogeli* from 0.6%. Regular antiparasitic treatments in dogs are important not only for preventing tick-infestation and canine TBPs, but especially in the context of public health. From a geographical point of view, our results confirm the widespread nationwide presence of piroplasms, with 68% of the sampled provinces positive for *Babesia* or *Theileria*. A higher prevalence of infection was reported in northern Italy (OR = 7.50, 95% CI 5.24–10.73), compared to central and southern provinces.

DNA of bacteria of the Anaplasmataceae family was reported in 46 of the sampled NUTS3 provinces (59% of the Italian territory included in the study) with an overall prevalence in tick pools of 10.5%. The highest infection prevalence was recorded in ticks from the NUTS3 in northern Italy, except for the province of Messina in Sicily, an area traditionally endemic for *Anaplasma* [35]. Here, 3 pools of *R. sanguineus* group were infected with *A. ovis*. Engorged females of *I. ricinus* were the most infected class of ticks, followed by *I. hexagonus*. *R. sanguineus* group was found to be infected with the highest variety of Anaplasmataceae species. *Anaplasma phagocytophilum* was the most widespread species detected in tick-pools positive at *Anaplasma*/*Ehrlichia* PCR and was detected with the highest MIR in *I. hexagonus* (MIR = 41.7%), followed by *I. ricinus* (MIR = 11.4%) and *R. sanguineus* group (MIR = 1.8%). *I. ricinus* is the primary vector of *A. phagocytophilum* in Europe, but the high infection rate of *I. hexagonus* confirms the important role that hedgehogs and hedgehog ticks may play in the epidemiology of *A. phagocytophilum* in Europe [36]. Previous studies report *A. phagocytophilum* in ticks of domestic dogs and wild carnivores from Italy, with a prevalence ranging from 0 to 16.6% [22, 37–45]. *A. platys* and *E. canis* were reported in tick pools from both northern and southern provinces (*p* > 0.05), in contrast with previous reports of higher seroprevalence levels in dogs from southern Italy [45, 46] and Sardinia [47]. Notably, *E. canis* DNA was detected in *R. sanguineus* group*,* which is its main tick vector in Mediterranean areas [48], but also with higher MIR in *I. ricinus* (OR = 15.15, 95% CI 3.47–66.16) and *I. hexagonus* (OR = 10.07, 95% CI 1.4–72.34).

*Borrelia burgdorferi* s.l. DNA was detected with low prevalence across the country, in both *I. ricinus* and *R. sanguineus* group. The geographical distribution of ticks infected with *B. burgdorferi s.l.* shows isolated infected tick pools from 8 of the 78 examined NUTS3 provinces, while in the province of Oristano (Sardinia) 2 tick pools from 2 different dogs were infected with *B. burgdorferi* s.l. A cross-sectional seroepidemiological study carried out in Sardinia [49] reported a seroprevalence of 6.1% in teen-agers but showed no association between seropositivity and pet ownership. In other Italian regions, anti-*B. burgdorferi* antibodies are present in the human population with a prevalence that varies considerably between geographical areas (from 0 to 23.2%) [50]. The results of our study confirm the localized distribution of *B. burgdorferi*, while the low number of ticks submitted from the northeastern regions of Italy (traditionally highly endemic for *B. burgdorferi* s.l.) [50] did not allow a detailed assessment of the epidemiological situation of dog-infesting ticks from this area.

*B. burgdorferi* s.l. DNA was detected in ticks infesting dogs exposed not only to rural and sylvatic environments, but also in ticks of dogs exposed to urban environments.

## Conclusions

The results obtained from this study highlight the high variability of piroplasms, Anaplasmatacea and Spirochaetae in dog-infesting ticks in Italy. Our data confirm that the emergence of TBPs, which have mainly wild reservoir hosts (i.e. roe deer for *B. venatorum* and wild rodents for *B. burgdorferi* s.s. and small mammals and wild ungulates for *A. phagocytophilum*) [9, 51–53], are not limited or confined to sylvatic and rural environments but are increasingly reported in anthropic biological communities (human, pet and, as in the present work, the ectoparasites of owned/pet dogs). The overall high prevalence of TBPs in ticks of privately-owned dogs reflects the importance of an in-depth understanding of ticks and TBPs by veterinary practitioners and veterinary authorities, which must duly inform pet owners and assist them in accessing preventive care through ectoparasitic treatments. A comparable extensive survey on TBPs infectious status of privately-owned dogs is greatly needed to complete the risk assessment of human exposure to zoonotic and tick-related infectious agents.

## Methods

### Sample collection and pathogen identification

A nationwide survey of ticks collected from privately-owned dogs in Italy was carried out over 20 months, from February 2016 to September 2017. The project involved 153 veterinary practices from 64 Italian provinces. Veterinarians were asked to check five randomly chosen dogs per month for ticks, and to complete a questionnaire for each dog. Each dog included in the study was only sampled once. The questionnaire requested information on date of sampling, geographical origin, breed, sex, age, coat length and ectoparasiticidal treatment history, housing and life environment. All collected ticks were morphologically identified at species level [54–56], and epidemiological risk factors as well as the owners’ habits regarding antiparasitic drug usage were evaluated, as reported by Maurelli et al. [23].

Results of morphological and molecular identification of the ticks analyzed in the present study has been previously reported [23]. We included in the present work only those tick species that are commonly reported to feed on dogs (Table 1). Identified ticks were divided into pools comprised of specimens collected from the same dog and homogeneous for species, developmental stage, sex and macroscopic engorgement status, then ginned with a sterile scalpel. The resulting material was homogenized in TRI-Reagent® (Sigma-Aldrich, Italy) and total DNA was extracted according to the manufacturer’s instructions with additional overnight incubation in Proteinase K (0.8 mg) and 500 μl of TRI-Reagent.

To detect *Babesia* spp. and *Theileria* spp., a semi-nested PCR targeting the V4 hypervariable region of the 18S rDNA using primers RLB-F2 (5′-GACACAGGGAGGTAGTGACAAG-3′), RLB-R2 (5′-CTAAGAATTTCACCTCTGACAGT-3′) and RLB-FINT (5′-GACAAGAAATAACAATACRGGGC-3′) was performed as described by [57]. For Anaplasmataceae, the 16S rDNA was targeted using primers PER1 (5′-TTTATCGCTATTAGATGAGCCTATG-3′) and PER2 (5′-CTCTACACTAGGAATTCCGCTAT-3′) [58]. *Borrelia burgdorferi* s.l. was detected using the primers FlaF (5′-AGAGCAACTTACAGACGAAATTAAT-3′) and FlaR (5′- CAAGTCTATTTTGGAAAGCACCTAA-3′), targeting a conserved region of the *fla* gene [59]. Positive (total genomic DNA from cultured parasites or confirmed clinical specimens) and negative controls (sterile bidistilled water) were included in each PCR reaction and all necessary measures were taken to minimize the risk of contamination. The PCR results were expressed as a minimum infection rate (MIR) or the minimum percentage of ticks in a pool with detectable DNA for each specific pathogen. This calculation was based on the assumption that a PCR-positive pool contains only one positive tick [60]. PCR-positive amplicons were purified using a commercial kit (Nucleospin Extract II Kit, Macherey-Nagel, Düren, Germany) and sequenced on both strands (Macrogen Europe, Spain) for species identification. The resulting nucleotide sequences were analyzed using MEGA X software [61] and compared to those available in GenBank (www.ncbi.nlm.nih.gov/genbank).

### Mapping and statistical analysis

Distributions of tick samples were geo-referenced using QGis [24], entering the owner’s hometown or, if missing, the location of the veterinary practice that enrolled the dog.

Chi-square tests, Odds ratio, logistic regressions and confidence intervals at 95% were calculated using R 3.4.4 [62]. Differences were considered significant at *p* < 0.05.

## Supplementary information

**Additional file 1: Table S1.** For each NUT3 (Province) where a minimum of 20 dogs was sampled, *Babesia/Theileria* Minimum Infection Rate (MIR) was calculated with Confidence Interval (CI) at 95%, Chi Square (χ2), Chi Square *p*-value (*significant values at *p* < 0.05) and Odds Ratio (with CI at 95%). **Table S2.** For each NUT3 (Province) where a minimum of 20 dogs had been sampled, *Anaplasma/Ehrlichia* Minimum Infection Rate (MIR) was calculated with Confidence Interval (CI) at 95%, Chi Square (χ2; calculated where applicable), Chi Square p-value (*significant values at p < 0.05) or Fisher Exact test *p* value (**significant values at p < 0.05) and Odds Ratio (with CI at 95%). **Table S3.** For each NUT3 (Province) where *Borrelia burgdorferi* s.l. was detected by PCR, we calculated Minimum Infection Rate (MIR) with Confidence Interval (CI) at 95%, Fisher Exact test p value (*significant values at p < 0.05) and Odds Ratio (with CI at 95%).

## Data Availability

All data generated and analyzed during this study are included in this published article and supplementary tables.

## Abbreviations

CI: Confidence Interval
LAU2: Local Administrative Units, level 2
MIR: Minimum Infection Rate
NUTS3: Nomenclature of Territorial Units for Statistics, level 3
OR: Odds Ratio
TBP: Tick-borne pathogen
