# Supplementary material for: Tick-borne pathogens in Ixodidae ticks collected from privately-owned dogs in Italy: a country-wide molecular survey
Source: BMC Vet Res. 2020 Feb 7;16:46. doi: 10.1186/s12917-020-2263-4 (PMC7006417; doi:10.1186/s12917-020-2263-4)
Supplement: Supplementary file 1 — Additional file 1: Table S1. For each NUT3 (Province) where a minimum of 20 dogs was sampled, Babesia/Theileria Minimum Infection Rate (MIR) was calculated with Confidence Interval (CI) at 95%, Chi Square (χ2), Chi Square p-value (*significant values at p < 0.05) and Odds Ratio (with CI at 95%). Table S2. For each NUT3 (Province) where a minimum of 20 dogs had been sampled, Anaplasma/Ehrlichia Minimum Infection Rate (MIR) was calculated with Confidence Interval (CI) at 95%, Chi Square (χ2; calculated where applicable), Chi Square p-value (*significant values at p < 0.05) or Fisher Exact test p value (**significant values at p < 0.05) and Odds Ratio (with CI at 95%). Table S3. For each NUT3 (Province) where Borrelia burgdorferi s.l. was detected by PCR, we calculated Minimum Infection Rate (MIR) with Confidence Interval (CI) at 95%, Fisher Exact test p value (*significant values at p < 0.05) and Odds Ratio (with CI at 95%). [file 12917_2020_2263_MOESM1_ESM.doc]

Additional file 1

**Table S1.** For each NUT3 (Province) where a minimum of 20 dogs was sampled, *Babesia/Theileria* Minimum Infection Rate (MIR) was calculated with Confidence Interval (CI) at 95%, Chi Square (χ2), Chi Square p-value (*significant values at p<0.05) and Odds Ratio (with CI at 95%).

| **Province - NUT3** | **Babesia/Theileria Positive** | **N. Tick pools** | **MIR** | **MIR 95%CI** | | ***χ*2** | **p-value** | **O.R.** | **O.R. 95%CI** | |
| --- | --- | --- | --- | --- | --- | --- | --- | --- | --- | --- |
| **Northern Italy** |  |  |  |  |  |  |  |  |  |  |
| Alessandria | 16 | 44 | 36.4% | 23.8% | 51.1% | 1.77 | 0.188 | 1.52 | 0.81 | 2.84 |
| Bergamo | 19 | 58 | 32.8% | 22.1% | 45.6% | 0.83 | 0.371 | 1.29 | 0.74 | 2.26 |
| Bologna | 13 | 21 | 61.9% | 40.9% | 79.3% | 12.60 | 0.000* | 4.37 | 1.8 | 10.6 |
| Cremona | 54 | 108 | 50.0% | 40.7% | 59.3% | 29.29 | 0.000* | 2.86 | 1.93 | 4.24 |
| Cuneo | 7 | 22 | 31.8% | 16.4% | 52.7% | 0.21 | 0.656 | 1.23 | 0.5 | 3.04 |
| Genova | 13 | 29 | 44.8% | 28.4% | 62. 5% | 4.43 | 0.036* | 2.17 | 1.03 | 4.55 |
| Milano | 30 | 56 | 53.6% | 40.7% | 66.0% | 19.71 | 0.000* | 3.18 | 1.86 | 5.45 |
| Novara | 4 | 22 | 18.2% | 7.3% | 38.5% | 0.98 | 0.319 | 0.58 | 0.2 | 1.72 |
| Padova | 4 | 55 | 7.3% | 2.9% | 17.3% | 11.72 | 0.001* | 0.2 | 0.07 | 0.55 |
| Parma | 28 | 77 | 36.4% | 26.5% | 47.5% | 3.16 | 0.000* | 1.54 | 0.95 | 2.48 |
| Pordenone | 6 | 28 | 21.4% | 10.2% | 39.5% | 0.54 | 0.461 | 0.71 | 0.29 | 1.77 |
| Reggio nell' Emilia | 16 | 51 | 31.4% | 20.3% | 45.0% | 12.52 | 0.000* | 3.6 | 1.69 | 7.67 |
| Treviso | 4 | 24 | 16.7% | 6.7% | 35.9% | 1.45 | 0.23 | 0.52 | 0.18 | 1.53 |
| Torino | 67 | 165 | 40.6% | 34.5% | 47.0% | 16.26 | 0.000* | 1.94 | 1.39 | 2.75 |
| Udine | 30 | 51 | 58.8% | 45.2% | 71.3% | 53.78 | 0.000* | 84.59 | 11.5 | 622.33 |
| Verona | 7 | 26 | 26.9% | 13.7% | 46.1% | 0.01 | 0.937 | 0.97 | 0.4 | 2.32 |
| Vicenza | 9 | 28 | 32.1% | 17.9% | 50.7 | 0.31 | 0.053 | 1.25 | 0.56 | 2.78 |
| **Central Italy** |  |  |  |  |  |  |  |  |  |  |
| Avellino | 2 | 21 | 9.5% | 2.7% | 28.9% | 3.46 | 0.062 | 0.27 | 0.06 | 1.18 |
| Campobasso | 1 | 25 | 4.0% | 0.7% | 19.5% | 7.05 | 0.008* | 0.11 | 0.01 | 0.8 |
| Frosinone | 2 | 24 | 8.3% | 2.3% | 25.8% | 4.50 | 0.033* | 0.24 | 0.06 | 1.01 |
| Latina | 4 | 43 | 9.3% | 3.7% | 21.6% | 7.36 | 0.006* | 0.26 | 0.09 | 0.74 |
| Pisa | 10 | 23 | 43.5% | 25.6% | 63.2% | 2.98 | 0.039* | 2.05 | 0.89 | 4.7 |
| Prato | 3 | 38 | 7.9% | 2.7% | 20.8% | 7.52 | 0.006* | 0.22 | 0.07 | 0.72 |
| Roma | 7 | 82 | 8.5% | 4.2% | 16.6% | 15.74 | 0.365 | 0.23 | 0.11 | 0.51 |
| **Southern Italy** |  |  |  |  |  |  |  |  |  |  |
| Caserta | 4 | 34 | 11.8% | 4.7% | 26.6% | 4.33 | 0.037* | 0.34 | 0.12 | 0.98 |
| Catania | 0 | 32 | 0.0% | 0.0% | 10.7% | 12.45 | 0.000* | 0 |  |  |
| Catanzaro | 3 | 53 | 5.7% | 1.9% | 15.4% | 13.18 | 0.000* | 0.15 | 0.05 | 0.49 |
| Messina | 0 | 22 | 0.0% | 0.0% | 14.9% | 8.47 | 0.004* | 0 |  |  |
| Napoli | 2 | 38 | 5.3% | 1.5% | 17.23% | 9.68 | 0.002* | 0.14 | 0.03 | 0.59 |
| Potenza | 0 | 22 | 0.0% | 0.0% | 14.9% | 8.47 | 0.004* | 0 |  |  |
| Siracusa | 0 | 25 | 0.0% | 0.0% | 13.3% | 9.65 | 0.002* | 0 |  |  |
| Taranto | 0 | 25 | 0.0% | 0.0% | 13.3% | 9.65 | 0.002* | 0 |  |  |

**Table S2.** For each NUT3 (Province) where a minimum of 20 dogs had been sampled, *Anaplasma/Ehrlichia* Minimum Infection Rate (MIR) was calculated with Confidence Interval (CI) at 95%, Chi Square (χ2; calculated where applicable), Chi Square p-value (*significant values at p<0.05) or Fisher Exact test p value (**significant values at p<0.05) and Odds Ratio (with CI at 95%).

| **Province- NUT3** | **Anap/Ehrl Positive** | **N. Tick pools** | **MIR** | **MIR 95%CI** | | **χ2** | **p-value** | **O.R.** | **O.R. 95%CI** | |
| --- | --- | --- | --- | --- | --- | --- | --- | --- | --- | --- |
| **Northern Italy** |  |  |  |  |  |  |  |  |  |  |
| Alessandria | 6 | 44 | 13.6% | 6.4% | 26.7% | 0.5 | 0.48 | 1.37 | 0.57 | 3.29 |
| Bergamo | 7 | 58 | 12.1% | 6.0% | 22.9% | 0.18 | 0.68 | 1.18 | 0.53 | 2.65 |
| Bologna | 0 | 21 | 0.0% | 0.0% | 15.5% |  | 0.15 | 0 |  |  |
| Cremona | 7 | 108 | 6.5% | 3.1% | 12.8% | 1.93 | 0.17 | 0.58 | 0.26 | 1.26 |
| Cuneo | 5 | 22 | 22.7% | 10.1% | 43.4% |  | 0.07 | 2.58 | 0.94 | 7.07 |
| Genova | 6 | 29 | 20.7% | 9.9% | 38.4% |  | 0.11 | 2.29 | 0.92 | 5.71 |
| Milan | 10 | 56 | 17.9% | 10.0% | 29.8% | 3.44 | 0.06 | 1.92 | 0.95 | 3.88 |
| Novara | 2 | 22 | 9.1% | 2.5% | 27.8% |  | 1 | 0.86 | 0.2 | 3.7 |
| Padova | 0 | 55 | 0.0% | 0.0% | 6.5% |  | 0.003** | 0 |  |  |
| Parma | 14 | 77 | 18.2% | 11.1% | 28.2% | 5.22 | 0.022* | 1.99 | 1.09 | 3.63 |
| Pordenone | 1 | 28 | 3.6% | 0.6% | 17.7% |  | 0.35 | 0.31 | 0.04 | 2.33 |
| Reggio nell' Emilia | 3 | 51 | 5.9% | 2.0% | 15.9% | 1.16 | 0.28 | 0.53 | 0.16 | 1.71 |
| Treviso | 3 | 24 | 12.0% | 4.9% | 26.5% |  | 0.74 | 1.12 | 0.33 | 3.78 |
| Torino | 28 | 165 | 17.0% | 12.7% | 22.3% | 8.25 | 0.004* | 1.9 | 1.22 | 2.96 |
| Udine | 9 | 51 | 17.7% | 9.6% | 30.3% | 2.5 | 0.09 | 1.88 | 0.9 | 3.94 |
| Verona | 2 | 26 | 7.7% | 2.1% | 24.1% |  | 1 | 0.71 | 0.17 | 3.04 |
| Vicenza | 2 | 28 | 7.1% | 2.0% | 22.7% |  | 0.76 | 0.66 | 0.15 | 2.79 |
| **Central Italy** |  |  |  |  |  |  |  |  |  |  |
| Avellino | 0 | 21 | 0.0% | 0.0% | 15.5% |  | 0.16 | 0 |  |  |
| Campobasso | 1 | 25 | 4.0% | 0.7% | 19.5% |  | 0.51 | 0.35 | 0.05 | 2.64 |
| Frosinone | 2 | 24 | 8.3% | 2.3% | 25.9% |  | 1 | 0.78 | 0.18 | 3.34 |
| Latina | 2 | 43 | 4.7% | 1.3% | 15.5% |  | 0.31 | 0.41 | 0.1 | 1.72 |
| Pisa | 2 | 23 | 8.7% | 2.4% | 26.8% |  | 1 | 0.82 | 0.19 | 3.51 |
| Prato | 4 | 38 | 10.5% | 4.2% | 24.1% |  | 1 | 1.01 | 0.35 | 2.89 |
| Roma | 2 | 82 | 2.4% | 0.7% | 8.5% | 5.9 | 0.015* | 0.20 | 0.05 | 0.84 |
| **Southern Italy** |  |  |  |  |  |  |  |  |  |  |
| Caserta | 3 | 34 | 8.8% | 3.1% | 23.0% |  | 1 | 0.83 | 0.25 | 2.74 |
| Catania | 0 | 32 | 0.0% | 0.0% | 10.7% |  | 0.1 | 0 |  |  |
| Catanzaro | 5 | 53 | 9.4% | 4.7% | 18.1% | 0.06 | 0.80 | 0.90 | 0.35 | 2.26 |
| Messina | 3 | 22 | 13.6% | 4.8% | 33.3% |  | 0.49 | 1.36 | 0.4 | 4.66 |
| Napoli | 2 | 38 | 5.3% | 1.5% | 17.3% |  | 0.42 | 0.47 | 0.11 | 1.97 |
| Potenza | 1 | 22 | 4.6% | 0.8% | 21.8% |  | 0.72 | 0.41 | 0.05 | 3.03 |
| Siracusa | 0 | 25 | 0.0% | 0.0% | 13.3% |  | 0.1 | 0 |  |  |
| Taranto | 1 | 25 | 4.0% | 0.7% | 19.5% |  | 0.16 | 0.35 | 0.05 | 2.64 |

**Table S3.** For each NUT3 (Province) where *Borrelia burgdorferi* s.l. was detected by PCR, we calculated Minimum Infection Rate (MIR) with Confidence Interval (CI) at 95%, Fisher Exact test p value (*significant values at p<0.05) and Odds Ratio (with CI at 95%).

| **Province- NUT3** | ***Borrelia* Positive** | **N. Tick pools** | **MIR** | **MIR 95%CI** | | **p-value** | **O.R.** | **O.R. 95%CI** | |
| --- | --- | --- | --- | --- | --- | --- | --- | --- | --- |
| **Northern Italy** |  |  |  |  |  |  |  |  |  |
| Cremona | 1 | 108 | 0.9% | 0.2% | 5.1% | 0.51 | 1.52 | 0.19 | 12.13 |
| Vicenza | 1 | 28 | 3.6% | 0.6% | 17.7% | 0.17 | 5.93 | 0.72 | 48.46 |
| **Central Italy** |  |  |  |  |  |  |  |  |  |
| Latina | 1 | 43 | 2.3% | 0.4% | 12.1% | 0.26 | 3.77 | 0.46 | 30.46 |
| **Southern Italy** |  |  |  |  |  |  |  |  |  |
| Caserta | 1 | 34 | 2.9% | 0.5% | 14.9% | 0.2 | 5.17 | 0.64 | 41.98 |
| Catanzaro | 1 | 53 | 1.9% | 0.4% | 8.0% | 0.29 | 3.24 | 0.4 | 26.04 |
| Medio Campidano | 1 | 13 | 7.7% | 1.4% | 33.3% | 0.08 | 14.41 | 1.69 | 122.78 |
| Messina | 1 | 22 | 4.7% | 0.8% | 21.8% | 0.13 | 8.19 | 0.99 | 67.54 |
| Oristano | 2 | 14 | 14.3% | 4.0% | 39.9% | 0.003* | 32.42 | 6.22 | 168.85 |
| Potenza | 1 | 22 | 4.6% | 0.8% | 21.8% | 0.131 | 8.19 | 0.99 | 67.54 |
